# Chemical Constituents from *Apios americana* and Their Inhibitory Activity on Tyrosinase

**DOI:** 10.3390/molecules23010232

**Published:** 2018-01-22

**Authors:** Jang Hoon Kim, Hyo Young Kim, Si Yong Kang, Jin-Baek Kim, Young Ho Kim, Chang Hyun Jin

**Affiliations:** 1 Advanced Radiation Technology Institute, Korea Atomic Energy Research Institute, Jeongeup, Jeollabuk-do 56212, Korea; oasis5325@gmail.com (J.H.K.); thy5012@kaeri.re.kr (H.Y.K.); sykang@kaeri.re.kr (S.Y.K.); jbkim74@kaeri.re.kr (J.-B.K.); 2 College of Pharmacy, Chungnam National University, Daejeon 34134, Korea; yhk@cnu.ac.kr

**Keywords:** *Apiosamericana*, Leguminosae, tyrosinase, slow binding inhibitor, molecular simulation

## Abstract

The goal of this study was to identify phytochemicals with inhibitory activity against tyrosinase. Nine compounds **1**–**9** were isolated from the tubers of *Apios americana.* This is the first report of aromadendrin 5-methyl ether (**1**) being isolated from the *Apios* species. Among them, compounds **2** and **8** showed inhibitory activity toward tyrosinase. Based on a Dixon plot, the potential *K*_i_ values of competitive inhibitors **2** and **8** were calculated as 10.3 ± 0.8 µM and 44.2 ± 1.7 µM, respectively. An IC_50_ value of 13.2 ± 1.0 µM was calculated for the slow-binding inhibitor **2** after preincubation with tyrosinase. Additionally, the predicted binding sites between the receptor and ligand, as well as secondary structure changes, in the presence of **2** were examined by molecular simulation.

## 1. Introduction

Tyrosinase (EC 1.14.18.1) is a copper-containing bifunctional enzyme found in plants, insects, and humans [1]. This enzyme is responsible for producing the necessary precursors during melanin biosynthesis [1]. In the presence of oxygen, tyrosinase catalyzes the hydroxylation of l-tyrosine to 3,4-dihydroxyphenylalanine (l-DOPA), followed by oxidation of l-DOPA to DOPA quinone [1,2]. Melanin protects human skin by absorbing UV radiation from sunlight, and it is also responsible for the browning of fruits, vegetables, and mushrooms [1,3]. However, this causes adverse effects, such as melisma, freckles and ephelis in skin [1,3]. Tyrosinase has been regarded as an enzyme target to attenuate these phenomena. Representative tyrosinase inhibitors, such as kojic acid and arbutin, have been developed for skin whiting from natural sources [4]. However, it has been reported that kojic acid has undesirable side effects, such as cytotoxicity, skin cancer, and dermatitis when used in cosmetics [5]. Recently, many studies has been performed to develop new tyrosinase inhibitor from natural plants [6,7,8].

Isoflavonoids are the naturally occurring main components of the Leguminosae family, which comprises ~19,500 species [9]. Isoflavonoids are secondary metabolites related to microorganisms in the roots and defense responses from outside pathogens [6] and are well-known phytoestrogens that are the binding into the estrogen receptor (ER) similar to 17-β-estradiol [10,11]. Among them, genistein was revealed to exhibit stronger affinity to the ER compared with 17-β-estradiol [10,11]. Isoflavonoids, which are the main phytoestrogen components of leguminous plants, are well-known ER antagonists [10,11,12]. Therefore, these compounds and estradiol were shown to play a role in melanin biosynthesis in normal human melanocytes in vitro [12].

*Apios americana* (*A. americana*), belonging to the Leguminosae family, is native to eastern North America [13,14]. The tubers of this plant were used as food sources by native American Indians and early European colonists [13,14]. *A. americana*, which is referred to as hodoimo or America-hodoimo, has been cultivated in Aomori Prefecture, Japan since the 19th century [15,16]. *Apios* powder has been used to make cookies, donuts, and bread [14]. Its tubers are reported to exhibit several biological activities (e.g., decreased blood pressure) [13]. Previous phytochemical studies of secondary metabolites have been performed on the tubers of *A. americana* [16]. From these studies, isoflavonoids such as 2′-hydroxygenistein and 2′-hydroxygenistein-7-*O*-glucoside, which suppress the binding of [^3^H]-dihydrotestosterone to their respective estrogen and androgen receptors, have been isolated [16,17].

The objective of this study was to isolate compounds from *A. americana* that regulate the catalytic reaction of tyrosinase in melanin biosynthesis. Isoflavonoids containing phytoestrogens were purified from the tubers of *A. americana* by column chromatography, and their inhibitory activity on tyrosinase was assessed in vitro. Furthermore, enzyme kinetics and molecular simulations were performed to gain insight into the enzyme/ligand complex.

## 2. Results and Discussion

### 2.1. Isolation and Identification

**Figure 1:**
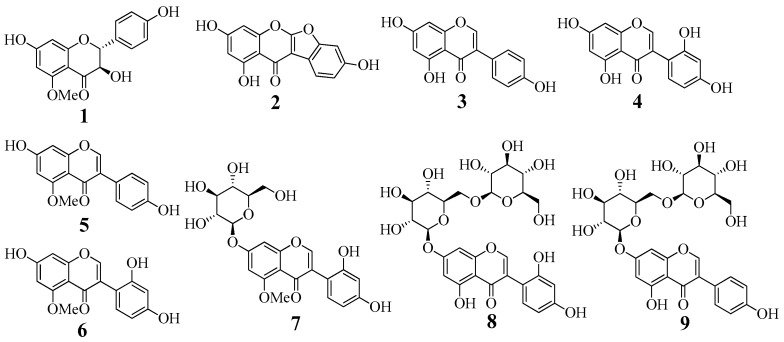
Structure of compounds **1**–**9** isolated from *A. americana*.

Dried tubers of *A. americana* were extracted with methanol at room temperature. The condensed methanol extract was suspended in water and progressively separated into *n*-hexane, ethyl acetate, butanol, and water fractions. The ethyl acetate fraction was subjected to silica gel, Sephadex LH-20, and C-18 column chromatography to furnish nine compounds **1**–**9**. Their structures were elucidated by comparing spectroscopic data (CD, ESI-MS, and NMR spectra) with those reported previously. The purified compounds were identified as aromadendrin 5-methyl ether (**1**) [18], lupinalbin A (**2**) [19], genistein (**3**) [17], 2′-hydroxygenistein (**4**) [17], 5-methylgenistein (**5**) [17], barpisoflavone (**6**) [17], 2′-hydroxy-5-methylgenistein-7-*O*-glucoside (**7**) [16], 2′-hydroxygenistein-7-*O*-gentibioside (**8**) [16], and genistein-7-*O*-gentibioside (**9**) [16] (Figure 1, Supplementary materials Figures S1–S11).

Interestingly, compound **1** was recently reported from the bark of *Akschindlium godefroyanum* and the roots of *Pyracantha coccinea* [18,20]. Our efforts have now led to isolation of compound **1** from the *Apios* species, which is reported for the first time.

### 2.2. Inhibitory Activity on Tyrosinase

To identify an effective inhibitor against tyrosinase, all nine isolated compounds **1**–**9** and eight reported compounds were screened in vitro using a UV-Vis method based on hydroxylation of l-tyrosine in the presence of tyrosinase [7]. The amount of l-DOPA converted from l-tyrosine as substrate of this enzyme was quantified in the presence of each respective compound [7]. Kojic acid derived from a natural source was used as a positive control [7].

**Figure 2:**
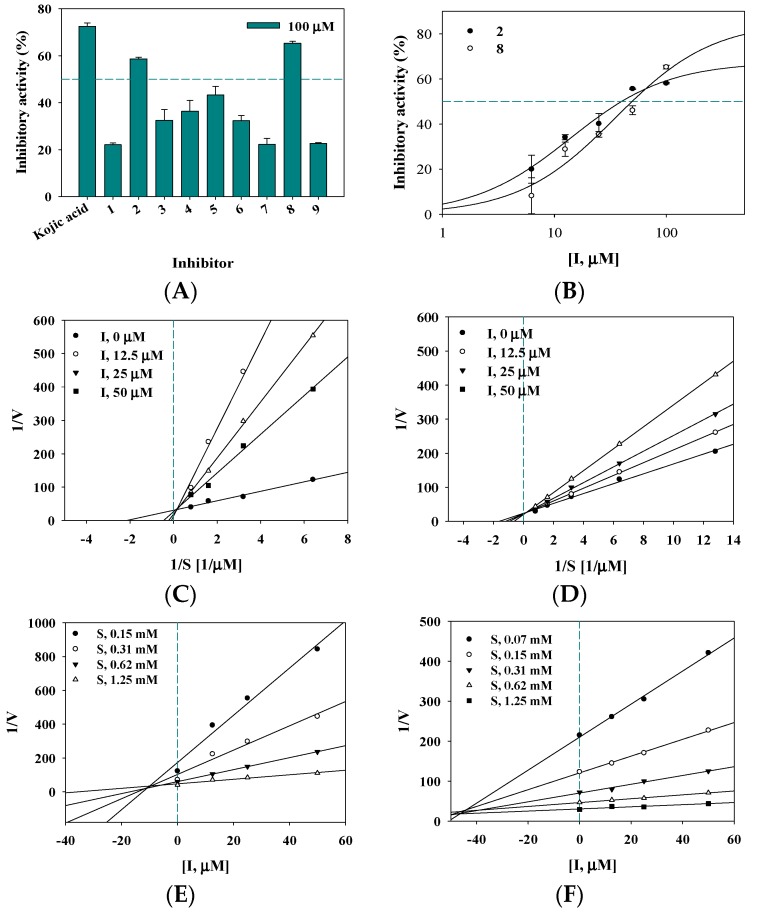
Inhibitory activity of compounds **1**–**9** (**A**) on tyrosinase and compounds **2** and **8** (**B**); Lineweaver-burk plot and Dixon plot of respective compounds **2** (**C**,**E**) and **8** (**D**,**F**).

Compounds **1**–**9** and eight reported compounds were evaluated for inhibition against tyrosinase at a concentration of 100 μM and exhibited inhibitory activity ranging from 22.1 ± 0.6 to 65.2 ± 0.8% of the control value (Figure 2A).

Of the tested compounds, **2** and **8** were evaluated at a concentration range of 6.2 to 100 µM to calculate their IC_50_ values. Both exhibited over 50% inhibitory activity in a dose-dependent manner, with IC_50_ values of 39.7 ± 1.5 µM and 50.0 ± 3.7 µM, respectively (Table 1 and Figure 2B).

According to a time-course enzyme assay, compound **2** interacted with tyrosinase in a time-dependent manner, whereas compound **8** did not exhibit this pattern. Additionally, preincubation of compound **2** with the enzyme for approximately 5 min exhibited inhibitory activity, with an IC_50_ value of 13.2 ± 1.0 µM.

The IC_50_ values for daidzein and genistein, the main isoflavonoids in soy constituents, in terms of tyrosinase inhibition were reported to exceed 500 µM [21]. Our study demonstrated IC_50_ values for compounds **2** and **8** of less than 50 µM; however, these compounds exhibited 0.5- and 0.6-fold less inhibitory activity, respectively, compared with the positive control. Interestingly, preincubation of the time-dependent inhibitor **2** with the enzyme increased the inhibitory activity on tyrosinase by 3- and 2-fold, respectively, compared with the IC_50_ values obtained in the absence of this preincubation and kojic acid.

### 2.3. Enzyme Kinetics

Enzyme kinetics are used to confirm the binding positions between an enzyme and inhibitor [7]. This study confirmed the effect of the substrate concentration on tyrosinase activity using four different concentrations [7]. The initial velocity (*v*_i_) was calculated at steady-state. A Lineweaver–Burk plot was represented by 1/*v* versus 1/substrate in the presence of inhibitor according to enzyme kinetics theory [7,22]. A series of regression lines for compounds **2** and **8** are presented in Figure 2C,D, respectively. Their regression lines intersected the *y*-axis at the same point and the *x*-axis at different points. These results confirmed a constant *V*_max_ with increasing *K*_m_ in the presence of different concentrations of the inhibitors (**2** and **8**) and showed that **2** and **8** disrupted the interaction between tyrosinase and its substrate in a competitive manner. These results are similar to those obtained using 6,7,4′-trihydroxyisolflavone, which was found to be a competitive inhibitor by Chang et al. [8]; its inhibitor constant (*K*_i_) value was obtained using a Dixon plot. The two inhibitors **2** and **8** examined herein exhibited *K*_i_ values of 10.3 ± 0.8 µM and 44.2 ± 1.7 µM, respectively (Figure 2E,F).

**Figure 3:**
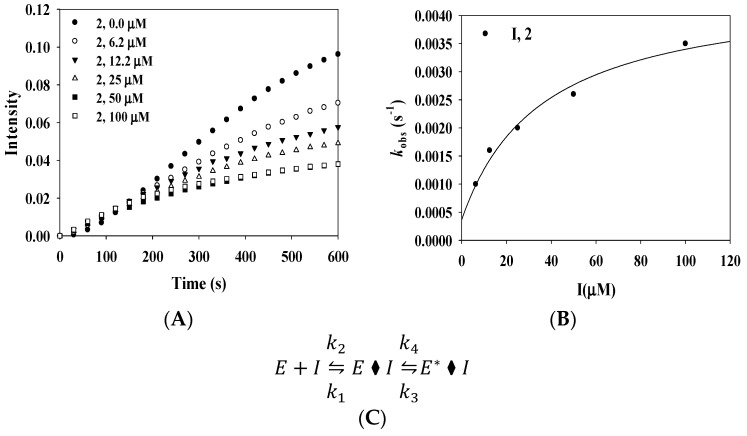
(**A**) Progress curves for slow-binding inhibition of tyrosinase by **2**; (**B**) Dependence of the values of *k_obs_* on the concentration of **2**; (**C**) Scheme for a slow-binding inhibition process.

In particular, compound **2** showed a typical slow-binding reaction progress curve for its catalytic reaction with tyrosinase. The *v*_i_ before equilibrium between the enzyme and ligand showed similar curves at different concentrations of **2** over a 150 s period, and the steady-state velocity (*v*_s_) decreased in a dose-dependent manner with increasing concentration of **2** (Figure 3A).

These curves were calculated by the following Equation (1):

(1) $$\left[P\right]=v_{s}t+\frac{v_{i}-v_{s}}{k_{obs}}\left[1-\exp\left(-k_{obs}t\right)\right]$$

where [P] is the concentration of product from the catalytic reaction, and the first-order rate constant (*k_obs_*) is the slow-binding constant corresponding to *v_i_* and *v_s_*.

The plot of *k_obs_* versus [inhibitor, *I*] is represented in Figure 3B. This result was incorporated into the following Equation (2):

(2) $$k_{obs}=k_{4}+\frac{k_{3}\left[I\right]}{K_{i}^{app}+\left[I\right]}$$

where $k_{3}$, $k_{4}$, and $K_{i}^{app}$ represent the kinetic parameters.

The resulting hyperbolic curve suggested that the enzyme binds to the ligand via a two-step process, as shown in Figure 3C. First, the initial enzyme–ligand complex (E·I) forms rapidly, and then the isomerized enzyme–ligand complex (E*·I) forms slowly. Using Equation (3), $k_{3}$, $k_{4}$, and $K_{i}^{app}$ kinetic parameters were calculated as 0.0041 S^−1^, 0.0004 S^−1^, and 35.8 µM, respectively (Table 2).

Finally, competitive inhibitor **2** was confirmed to inhibit tyrosinase in a time-dependent manner.

### 2.4. Molecular Docking

**Figure 4:**
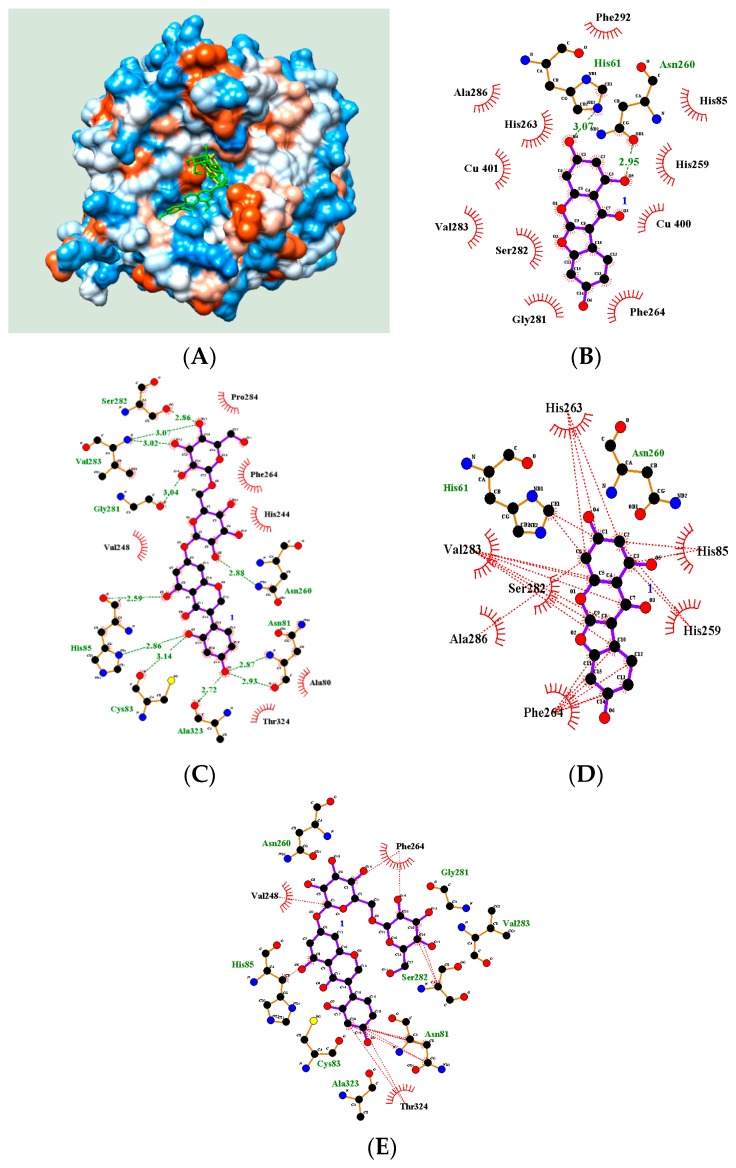
Predicted docking poses of **2** (yellow) and **8** (green) with enzyme (**A**); The green arrow represented hydrogen bond (**B**,**C**) and hydrophobic (**D**,**E**) interactions between respective ligands (**2** and **8**) and receptor.

Computer simulation helps determine potential inhibitors of a particular enzyme [22]. Our previous studies used molecular docking based on enzyme assays to show how inhibitors anchor to an enzyme [7,22,23]. In the current study, molecular docking was performed to examine the enzyme–inhibitor complex using AutoDock 4.2. The 3D structure of tyrosinase was adopted from the RCSB PDB (PDB ID: 2Y9X) [7]. Ligands **2** and **8** showed a familiar active site according to enzyme kinetics. As reported previously, a grid was set up in the space containing two copper ions [18]. Molecular simulation was performed 25,000,000 times, and the complex with the lowest AutoDock score was considered to represent the best docking pose [18,22,24]. As shown in Figure 4A, two ligands, **2** and **8**, were appropriately docked in the active site with AutoDock scores of −5.87 and −4.58 kcal/mol, respectively (Table 3).

Inhibitor **2** was docked in the active site via hydrogen bonds with His61 and Asn260 of 3.07 Å and 2.95 Å, respectively (Figure 4B). Figure 4D shows the hydrophobic interactions between inhibitor **2** and eight amino residues (His61, His85, His259, His263, Phe264, Ser282, Val283, and Ala286) in the active site. However, inhibitor **8** bound to the active site, and adjacent cavity via eight hydrogen bonds (Ala81: 2.87, 2.93; Cys83: 3.14; His85: 2.59, 2.86; Gly281: 3.04; Ser282: 2.86; Val283: 3.02, 3.07; Asn260: 2.88; and Ala323: 2.72 Å) and hydrophobic interactions with six amino acids (Asn81, His85, Val248, Phe264, Ser282, and Thr324) (Figure 4C,E). The inhibitor **8** with over 500 molecular weight indicated an additional interaction at the cavity next to the active site. Our previous report also revealed a similar binding pattern [7].

### 2.5. Molecular Dynamics

**Figure 5:**
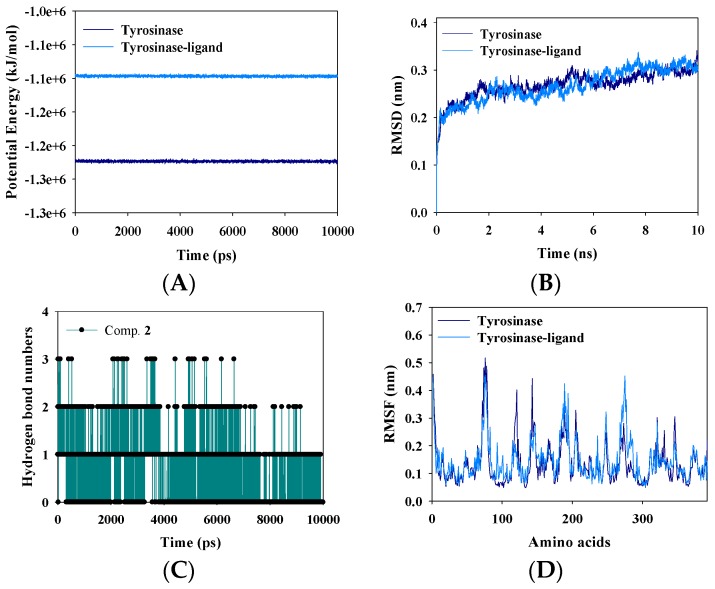
The potential energy (**A**); RMSD (**B**); Hydrogen bonding number (**C**); and RMSF (**D**) of tyrosinase without ligand and tyrosinase with **2**, respectively.

MD is a state-of-the-art virtual aid for analyzing the flexibility of a receptor–ligand complex over time. The Gromacs 4.6.5 program was employed to simulate the respective tyrosinase and tyrosinase–ligand complex. Both were stably simulated (over 10 ns) with potential energy values of −1.25 × 10^6^ and −1.1 × 10^6^ kJ/mol, respectively (Figure 5A). The latter exhibited a higher potential energy than that of the former because of the interaction between tyrosinase and the ligand. The potential energy was calculated using the root-mean-square deviations (RMSD) under a distance of approximately 0.3 nm (Figure 5B). Furthermore, hydrogen bonds are important for the interaction between a receptor and ligand. Compound **2** interacted mostly with one or two residues in tyrosinase and sometimes formed four or no hydrogen bonds for the trajectory period of the MD simulations (Figure 5C).

**Figure 6:**
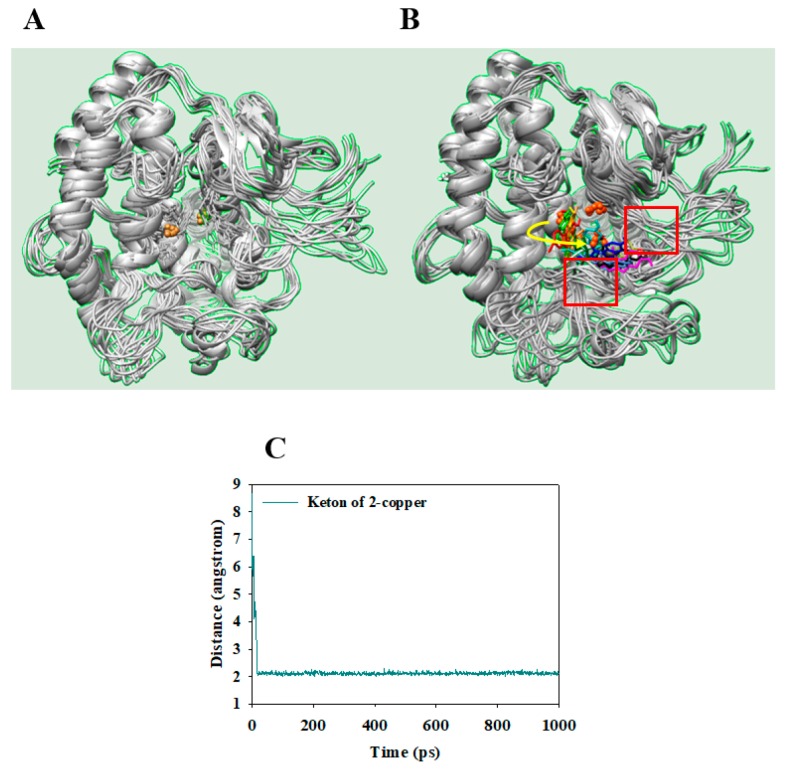
The superpositions of apo-receptor (**A**) and receptor with ligand (**B**) for the simulation time (red: 0, orange: 1, yellow: 2, green: 3, forestgreen: 4, cyan: 5, blue: 6, purple: 7, hot pink: 8, magenta: 9, and, black:10 during 10 ns). The distance of copper with ligand (**C**).

Additionally, to determine the effect of the ligand on residue mobility and the structure of the two receptors, tyrosinase and the tyrosinase–ligand complex were analyzed using the root-mean-square fluctuation (RMSF) MD simulation was performed using the above mentioned parameters to determine the overlapping secondary structures and position of copper ions in apo-tyrosinase (Figure 6A). The ligand binding to tyrosinase restricted the fluctuation of the receptor, as this flowed into the interspace between two loops represented as red box (Figure 6B). Compound **2** had little impact on the three histidines bound to copper. However, the ketone of **2** maintained a 2 Å distance with a copper ion for 10,000 ps (Figure 6C); this removed one copper ion from a deep cavity within the active site. These results indicate a potentially new enzyme conformation isomerized by a slow-binding inhibitor.

## 3. Materials and Methods

### 3.1. General Experimental Procedures

Optical rotation was measured using a JASCO P-1020 polarimeter (Easton, MD, USA), and circular dichroism (CD) spectra were recorded on a JASCO J-600 spectrometer. Column chromatography was performed using silica gel (Kieselgel 60, 70–230, and 230–400 mesh, Merck, Darmstadt, Germany), Sephadex LH-20 (GE Healthcare, Uppsala, Sweden), and C-18 resins. Thin-layer chromatography was performed using pre-coated silica gel 60 F_254_ and RP-18 F_254S_ plates (both 0.25 mm, Merck). Spots were visualized by spraying with 10% aqueous H_2_SO_4_ solution followed by heating. Nuclear magnetic resonance (NMR) spectra were recorded using an ECA 500 spectrometer (^1^H, 500 MHz; ^13^C 125 MHz, JEOL, Tokyo, Japan). Mass spectra were measured using the Agilent LC-MS 6100 (SCL; Santa Clara, CA, USA). Tyrosinase (T3824), L-tyrosine (T3754), and kojic acid (K3125) were purchased from Sigma-Aldrich (St. Louis, MO, USA).

### 3.2. Plant Materials

Tubers of *A. americana* were cultivated and collected at the Radiation Breeding Research Center (RBRC) and Korea Atomic Energy Research Institute (KAERI) in October 2016 and identified by Dr. S. Y. Kang at KAERI. A voucher specimen (RBRC001) was deposited at the herbarium of RBRC.

### 3.3. Extraction and Isolation

Dried tubers of *A. americana* (4 kg) were extracted three times for 1 week at room temperature with 95% methanol (4 L). The crude extract (337 g) condensed under reduced pressure was dissolved in water (3 L). The suspended extract was partitioned using *n*-hexane, ethyl acetate, butanol, and water, in succession. The ethyl acetate fraction (9 g) was separated by silica gel column chromatography using a chloroform/methanol gradient system (30/1→3/1) to yield twelve fractions E1–12. Fraction E5 was subjected to C-18 column chromatography using a gradient elution of water/methanol (2/1→1/5) to obtain compound **1** (4 mg) and four fractions E51–54. Fraction E53 was subjected to Sephadex LH-20 column chromatography using 95% methanol to yield compounds **6** (17 mg) and **2** (12 mg). The E54 fraction was separated by Sephadex LH-20 column chromatography using 95% methanol to obtain two fractions E541 and E542. E541 was purified by C-18 column chromatography using an isocratic system of 65% methanol to yield compound **5** (15 mg). The E7 fraction was subjected to C-18 column chromatography using a gradient elution of water/methanol (3/1→1/3) to furnish six fractions E71–E76. Compounds **3** (8 mg) and **4** (14 mg) were purified from the E72 fraction by Sephadex LH-20 column chromatography using an isocratic system of 60% methanol. The E74 fraction was separated by C-18 column chromatography using a water/methanol gradient system (5/1→0.5/1) to obtain compound **7** (5 mg). The E10 fraction was subjected to C-18 column chromatography using a gradient elution of water/methanol (10/1→1/1) to yield five fractions (E101–105). E101 was subjected to C-18 column chromatography using 80% methanol to yield compound **9** (7 mg). The E103 fraction was loaded onto Sephadex LH-20 column and eluted with methanol to afford compound **8** (9 mg).

### 3.4. Enzyme Inhibition Assay

The tyrosinase inhibition assay was performed as described by Kim et al. [7]. Briefly, tyrosinase (~45 U/mL, 130 µL) in 50 µM phosphate buffer (pH 6.8) was added to 96-well plates. Twenty microliters of each respective compound (1 mM) were dissolved in MeOH, and compounds **2** and **8** (diluted to 0.065 mM) were added. Finally, 50 µL 1.5 mM l-tyrosine were added as a substrate. After initiating the enzyme reaction at 37 °C, the products were measured using UV-Vis determination (wavelength, 475 nm) for 20 min. The inhibition ratio was calculated using the following Equation (3):

Inhibitory activity (%) = [(*Δ*control − *Δ*inhibitor)/*Δ*control] × 100 (3)

### 3.5. Molecular Simulations

Molecular docking was performed using the AutoDock 4.2 program as described previously [23,24]. The three-dimensional (3D) structure of the ligand was constructed using Chem3D Pro. Its flexible bonds were determined using AutoDockTools. The tyrosinase structure (PDB ID: 2Y9X) was obtained from Research Collaboratory for Structural Bioinformatics Protein Data Bank (RCSB PDB). Next, holmium, tropolone, and water in the receptor were removed with the exception of two copper ions. Hydrogens were added to tyrosinase using AutoDockTools.

### 3.6. Molecular Dynamics

Gromacs version 4.6.5 package was employed for the molecular dynamics (MD) simulation of tyrosinase and the tyrosinase–ligand complex. Molecular simulations were performed as described previously [24,25]. Tyrosinase was assigned by the Gromos96 43a1 force field for MD simulation. Additionally, gro and itp files for the ligand were produced using the prodrg server and this complex was solved with water in the cubic box with dimensions of 9.5 × 9.5 × 9.5 using the simple point charge water model. Furthermore, mdp files were built using the Gromacs homepage. Sodium ions were then added and minimized until a maximal force of 10 kJ/mol was reached using the steepest descent method. The minimized complex was equilibrated under 300 K conditions for 100 ps. The product equilibrated by Number Volume Temperature (NVT) was further performed using 1 bar Number Pressure Temperature (NPT) for 100 ps. The MD simulation was carried out for 10,000 ps.

### 3.7. Statistical Analysis

All tests in the presence of inhibitors were performed in triplicate. Results are presented as means ± standard error of the mean (SEM). The data were analyzed using Sigma Plot (SPP Inc., Chicago, IL, USA).

## 4. Conclusions

We aimed to isolate isoflavonoids from *A. americana* using column chromatography and to elucidate the structures of nine compounds **1**–**9**. Of these, flavanone **1** was reported for the first time in the *Apios* species. To confirm their biological effects in vitro, all compounds were tested for their inhibition of tyrosinase catalytic activity. Compounds **2** and **8** had IC_50_ values of 39.7 ± 1.5 µM and 50.0 ± 3.7 µM, respectively. To gain insight on the receptor–ligand complex, we performed enzyme kinetics and molecular simulations. The former indicated that the two compounds act as competitive inhibitors, with *K*_i_ values of 10.3 ± 0.8 µM and 44.2 ± 1.7 µM, respectively. The slow-binding inhibitor (**2**) had an IC_50_ value of 13.2 ± 1.0 µM after preincubation with tyrosinase. By classical enzymatic scholar theory, the grid for docking of competitive inhibitors was set up within the active site with two coppers, and then the two inhibitors were docked into the active site of the receptor. Based on these results, two inhibitors **2** and **8** showed corresponding AutoDock scores of −5.87 and −4.58 kcal/mol, respectively, compared with enzyme inhibitory activity. In particular, the potential slow-binding inhibitor **2** interacted with two loops of residues (50–65 and 275–290) an the molecular dynamics timescale. Moreover, this interaction affected the important copper ion, located in three pairs of histidines, necessary for catalysis. Overall, our study suggests that phytoestrogen **2** from *A. americana* acts as a tyrosinase inhibitor.

## Figures and Tables

**Table 1:** Tyrosinase inhibitory activities of compounds **2** and **8** from *A. americana.*

	Inhibitory Activity on Tyrosinase	
	IC_50_ (μM) ^a^	Binding Mode (*K*_i_: μM) ^a^
2	39.7 ± 1.5 13.2 ± 1.0 ^c^	Competitive (10.3 ± 0.8)
8	50.0 ± 3.7	Competitive (44.2 ± 1.7)
Kojic acid ^b^	25.2 ± 0.8	

^a^ All compounds were tested in a set of triplicated experiment. ^b^ Positive control. ^c^ IC_50_ value of preincubated inhibitor with enzyme.

**Table 2:** Kinetics parameters of the time-dependent tyrosinase inhibitory activity exhibited by **2**.

	*k*_5_(S^−1^)	*k*_6_(S^−1^)	$K_{i}^{\mathrm{app}}$(μM)
2	0.0041	0.0004	35.8

**Table 3:** Interaction and Autodock scores between tyrosinase and compounds **2** and **8**.

	Hydrogen Bonds (Å)	Binding Energy (kcal/mol)
2	His61(3.07), Asn260(2.95)	−5.87
8	Asn81(2.87, 2.93), Cys83(3.14), His85(2.86, 2.59), Gly281(3.04), Val283(3.02, 3.07), Ser282 (2.86), Asn260(2.88)	−4.58

## Supplementary Materials

Supplementary materials are available online.
