# Supplementary material for: Chemical Constituents from Apios americana and Their Inhibitory Activity on Tyrosinase
Source: Molecules. 2018 Jan 22;23(1):232. doi: 10.3390/molecules23010232 (PMC6017567; doi:10.3390/molecules23010232)
Supplement: Supplementary file 1 [file molecules-23-00232-s001.pdf]

Chemical constituents from *Apios americana* and their inhibitory activity on tyrosinase

Jang Hoon Kim[a], Hyo Young Kim[a], Si Yong Kang[a], Jin-Baek Kim[a],

Young Ho Kim[b], Chang Hyun Jin[a,]**

[a]*Advanced Radiation Technology Institute, Korea Atomic Energy Research Institute, Jeongeup, Jeollabuk-do 56212, Republic of Korea*

[b]*College of Pharmacy, Chungnam National University, Daejeon 34134, Republic of Korea*

*Corresponding author

Tel.:+82 63 570 3162 (C.H. Jin); fax:+82 63 570 3159 (C.H. Jin); e-mail address: chjin@kaeri.re.kr (C.H. Jin)

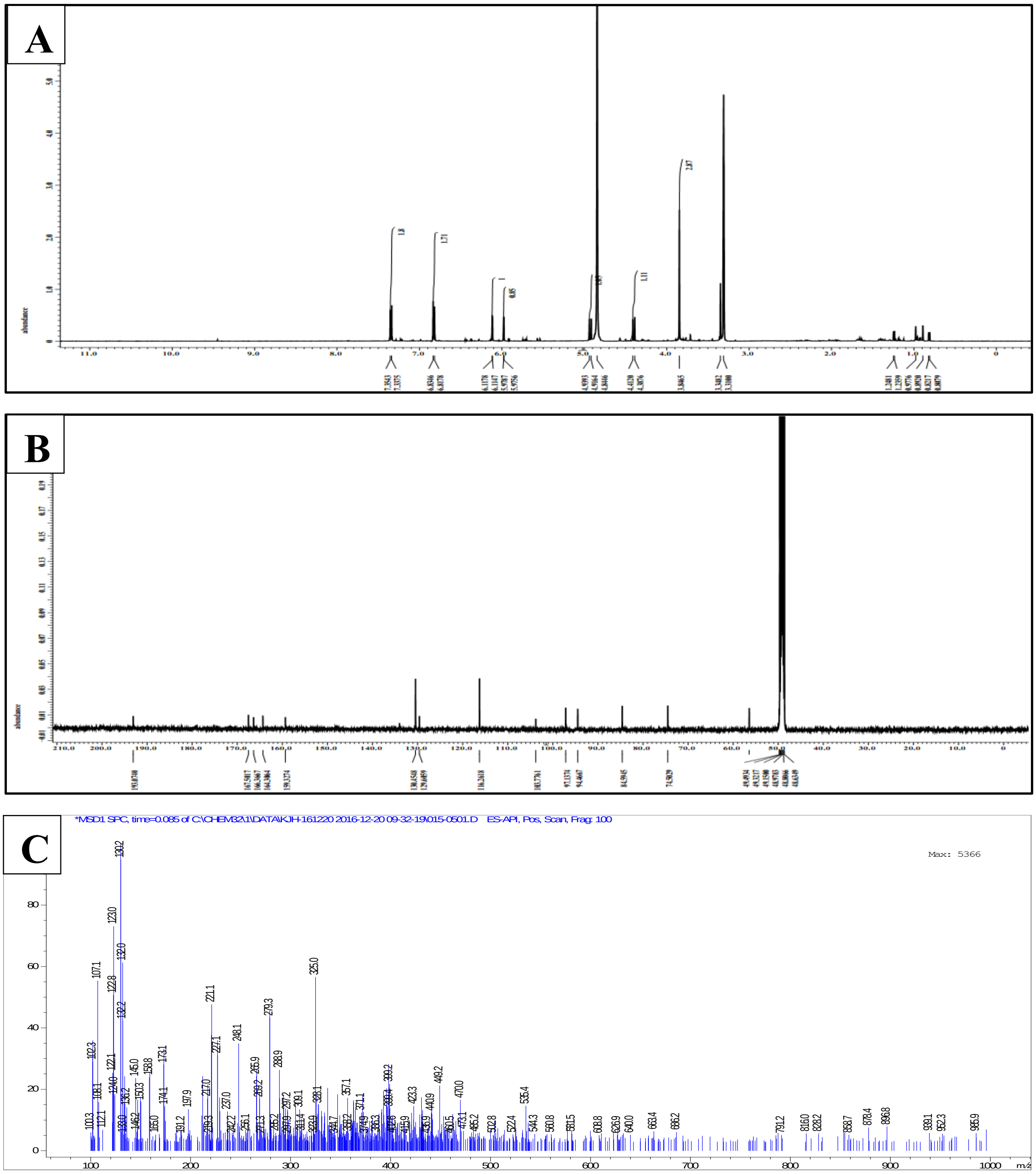

Figure S1. ^{1}H(A)-/^{13}C(B)-NMR and MS(C) spectra of compound **1**.

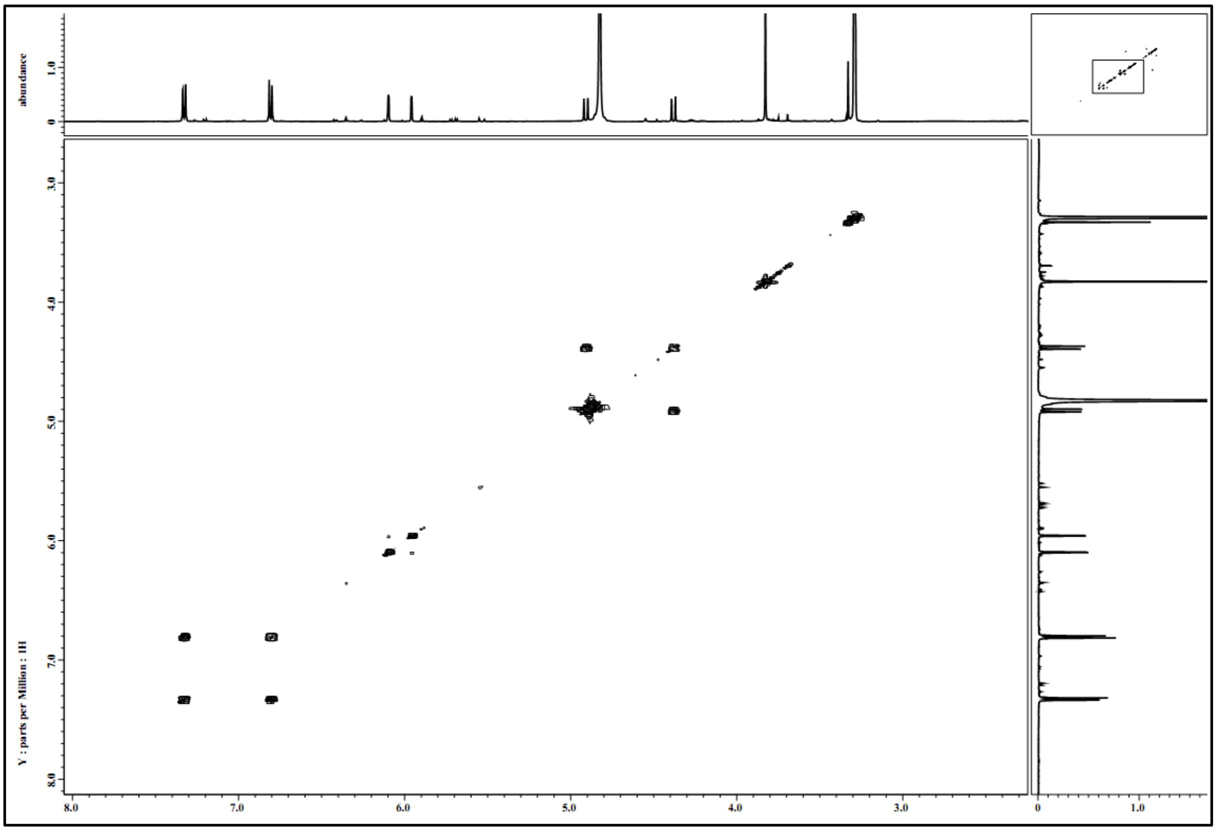

Figure S2. COSY of compound **1**.

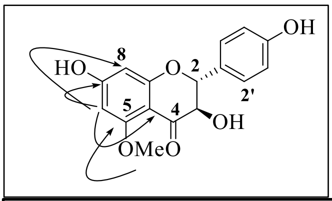

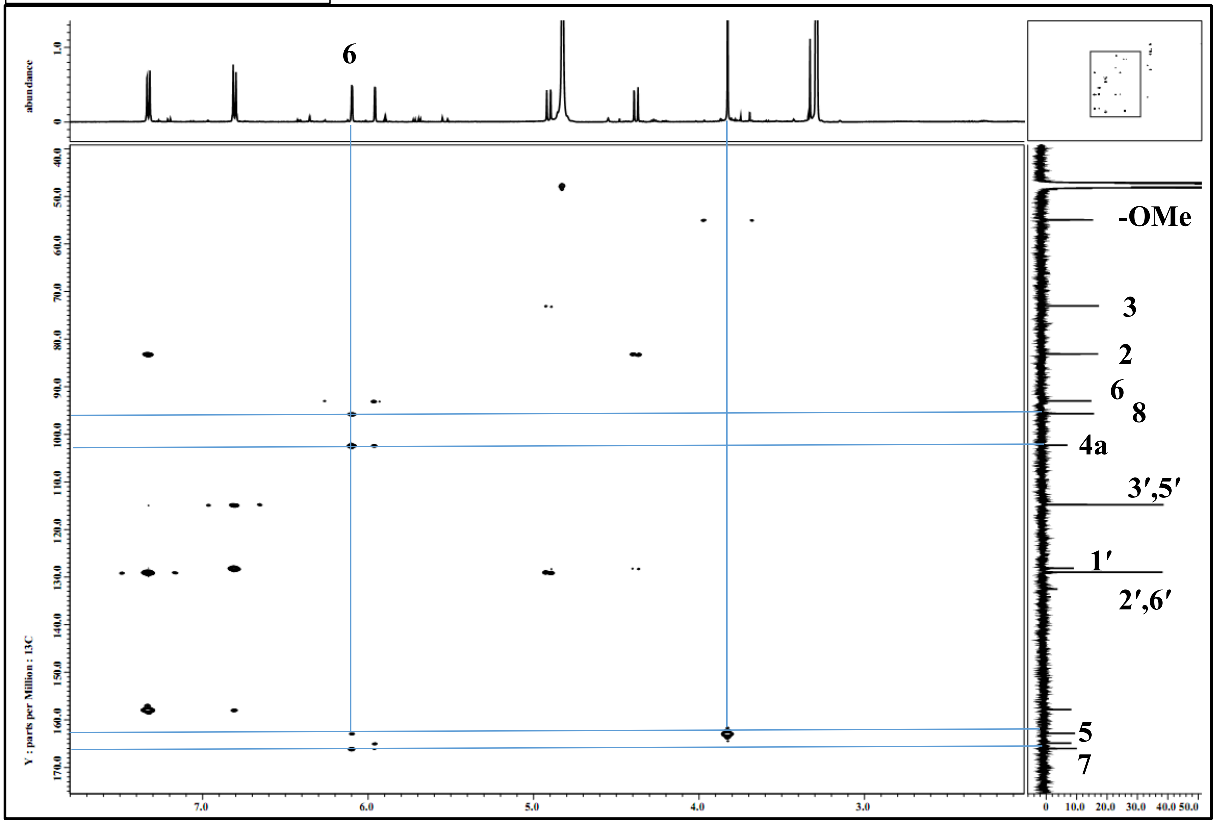

Figure S3. HMBC of compound **1**.

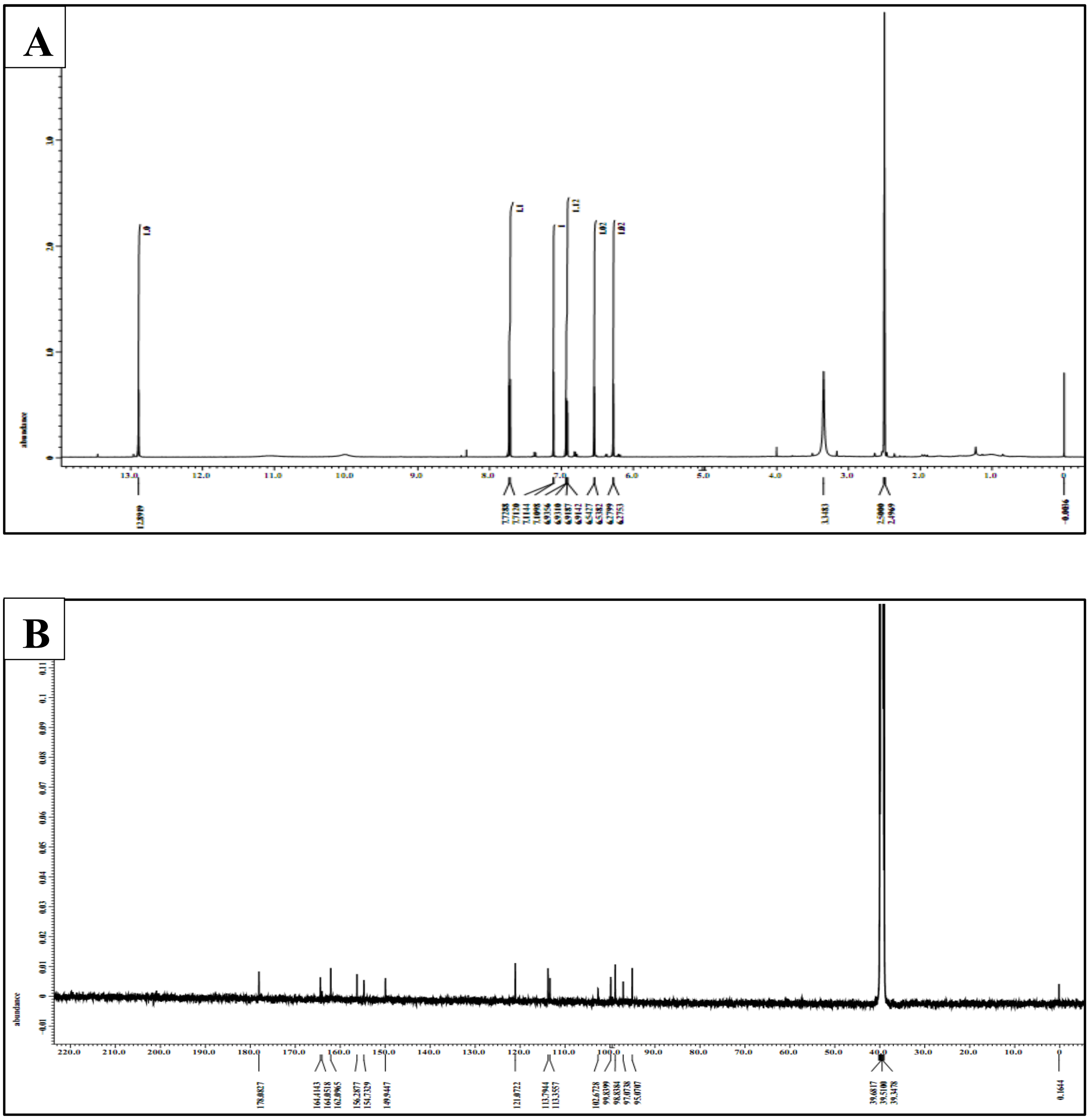

Figure S4. ^{1}H(A) and ^{13}C(B)-NMR of compound **2**.

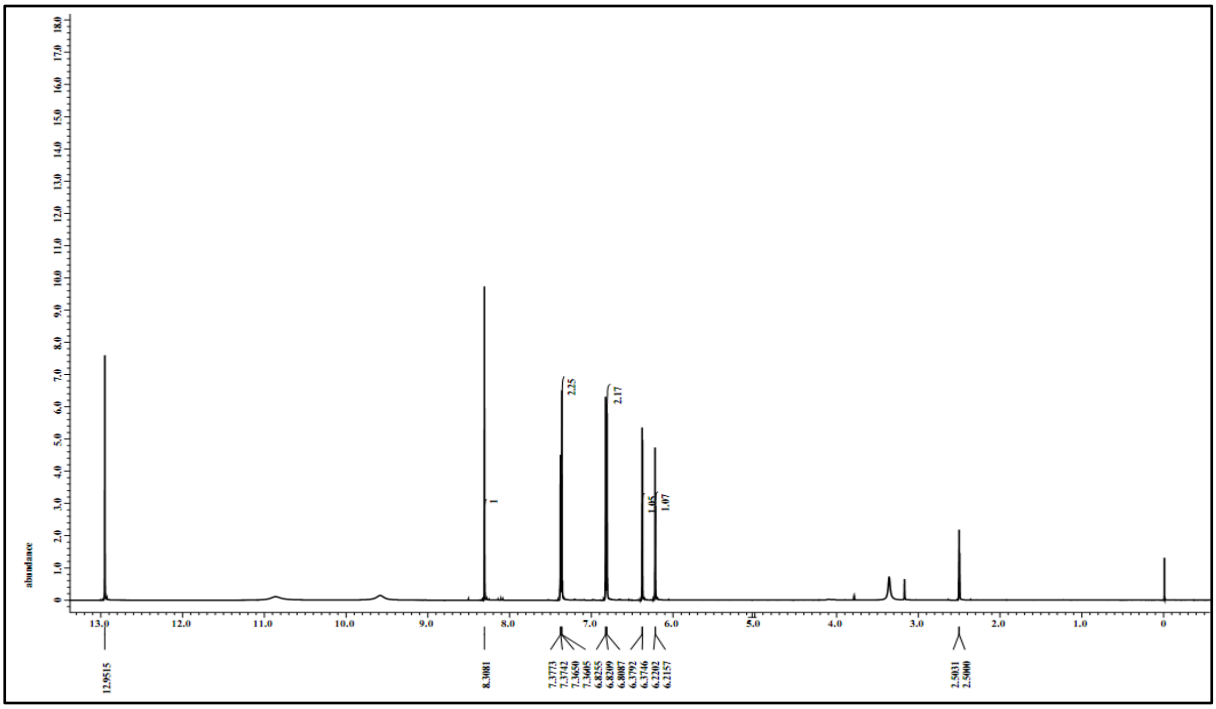

Figure S5. ^{1}H-NMR of compound **3**.

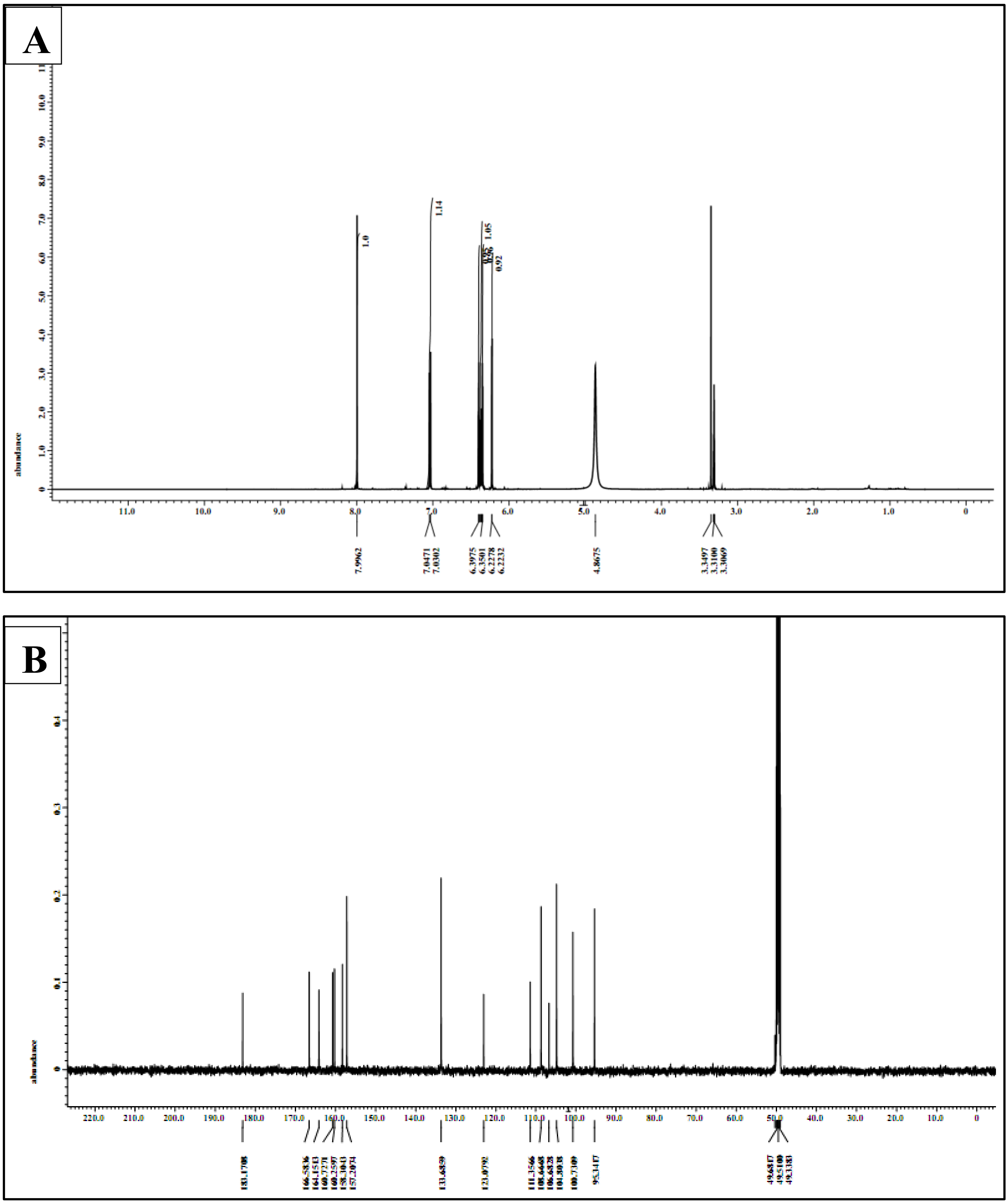

Figure S6. ^{1}H(A) and^{13}C(B)-NMR of compound **4**.

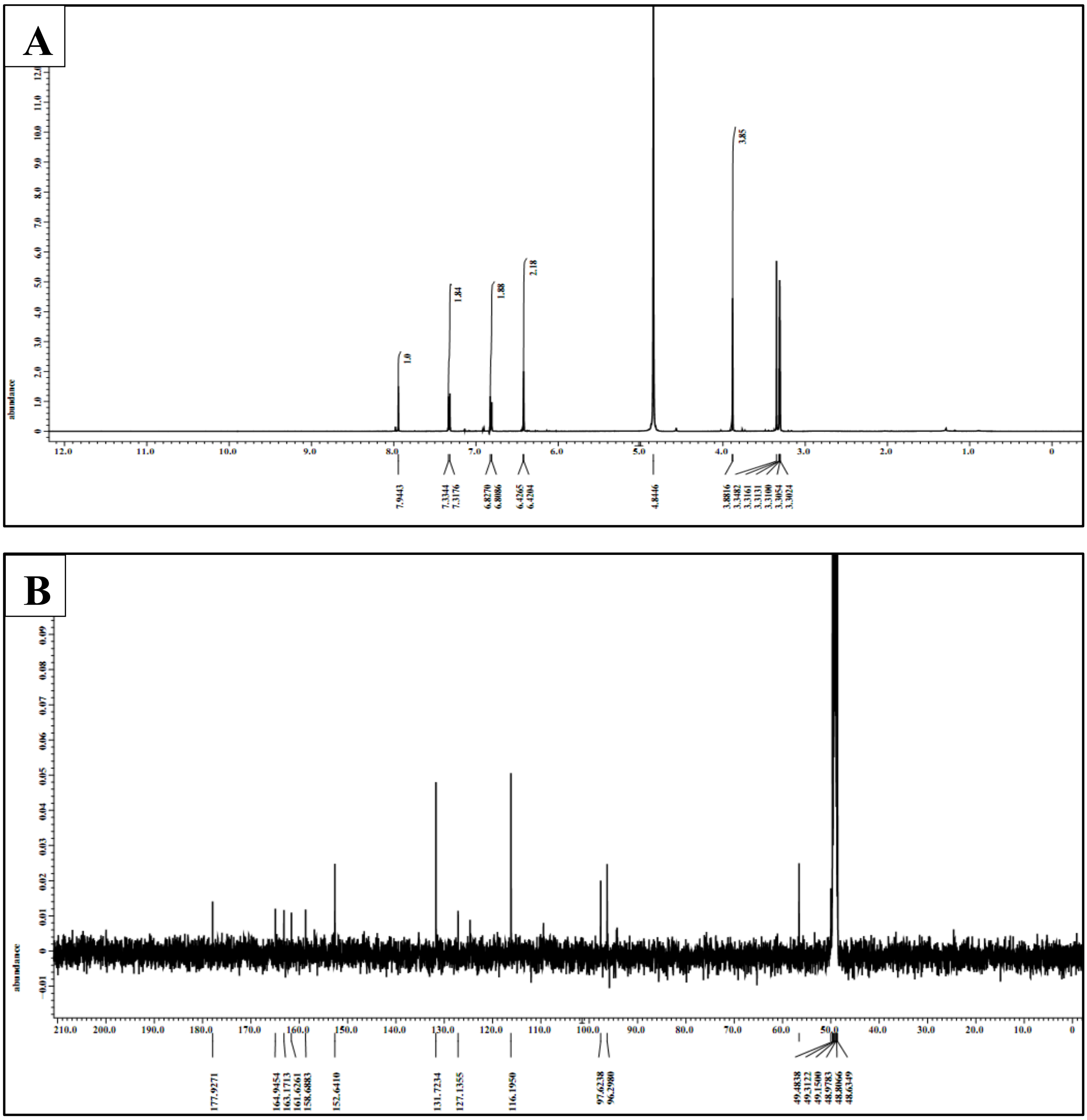

Figure S7. ^{1}H(A) and ^{13}C(B)-NMR of compound **5**.

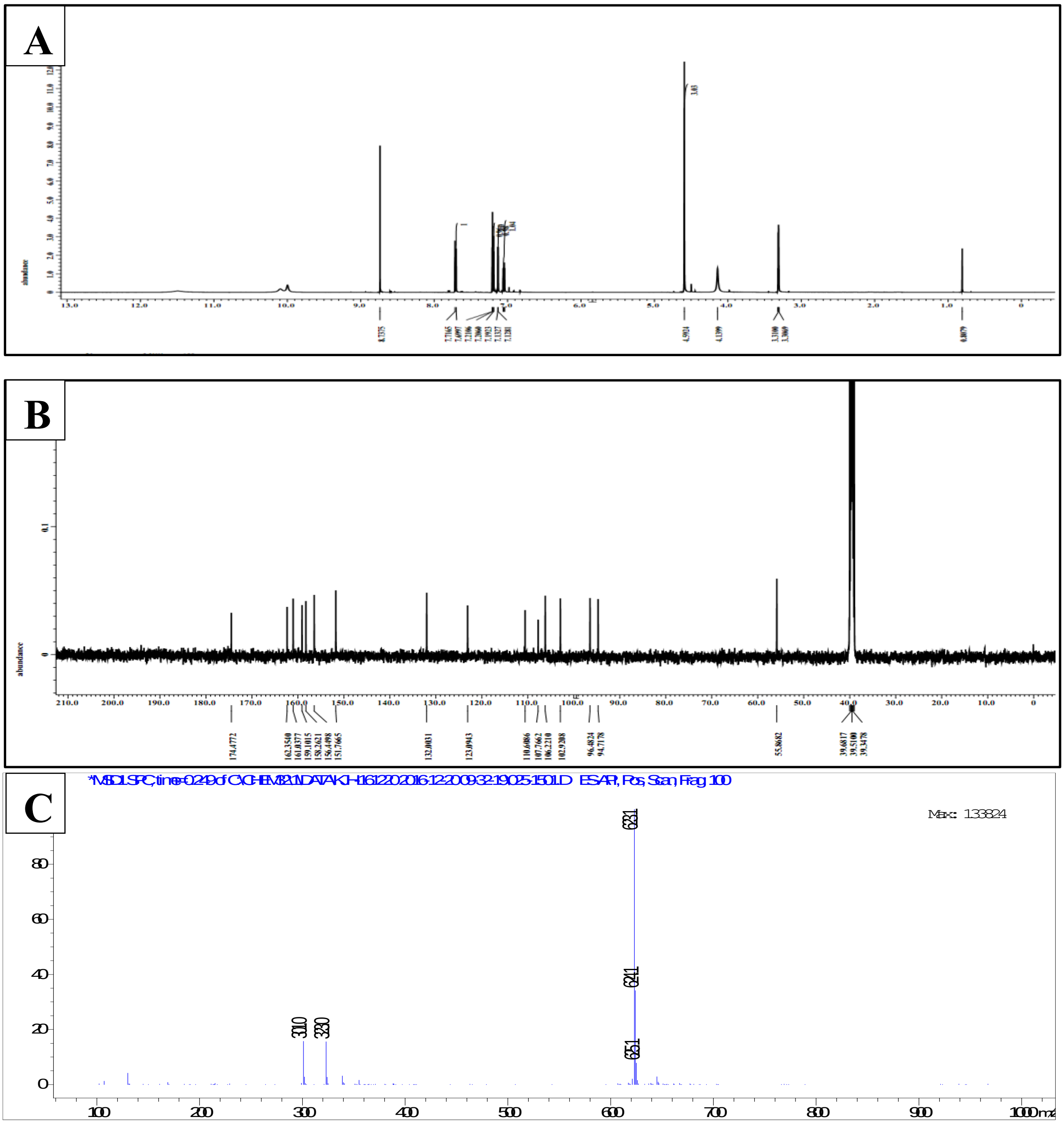

Figure S8. ^{1}H(A) and^{13}C(B)-NMR of compound **6**.

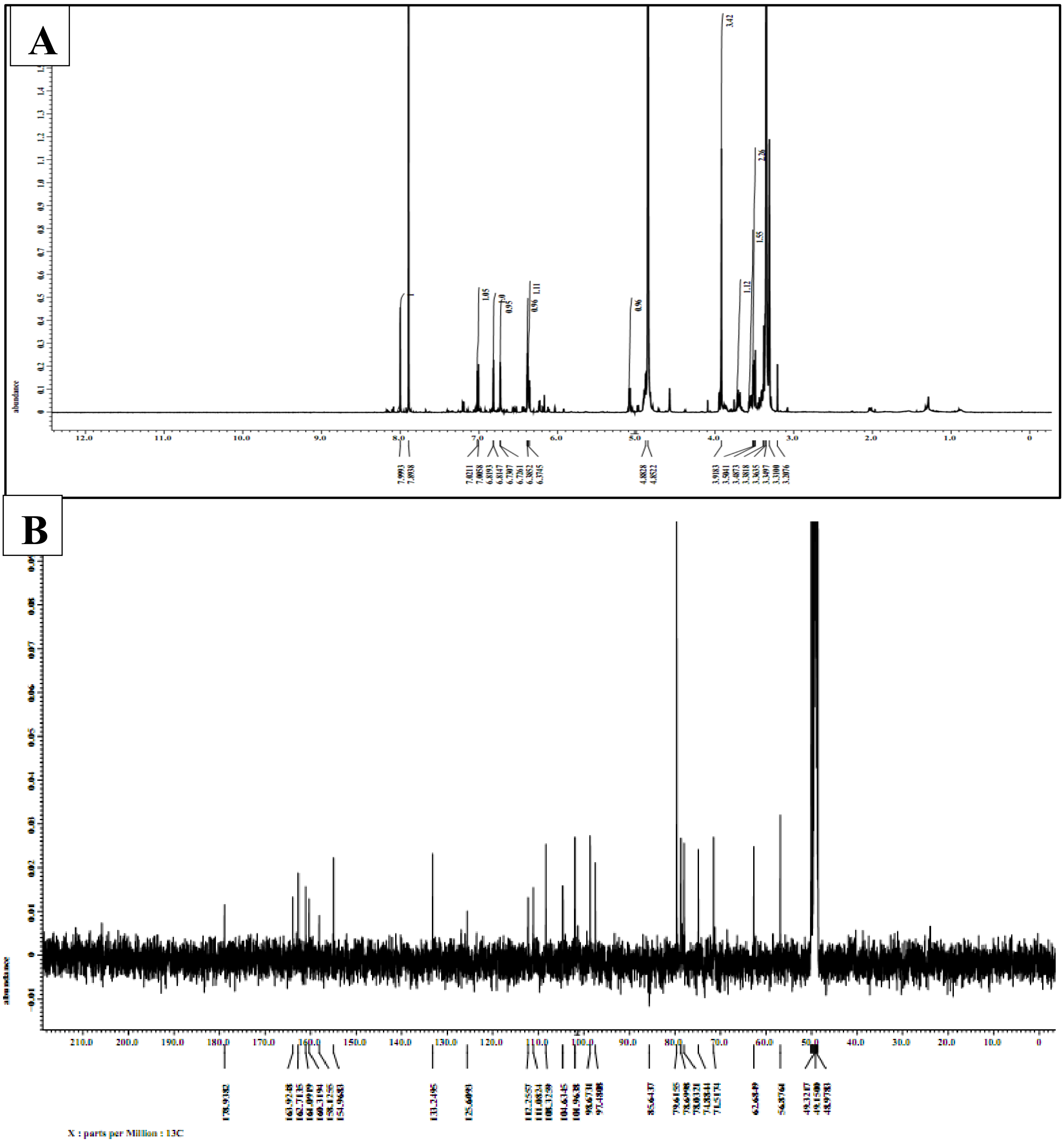

Figure S9. ^{1}H(A) and^{13}C(B)-NMR of compound **7**.

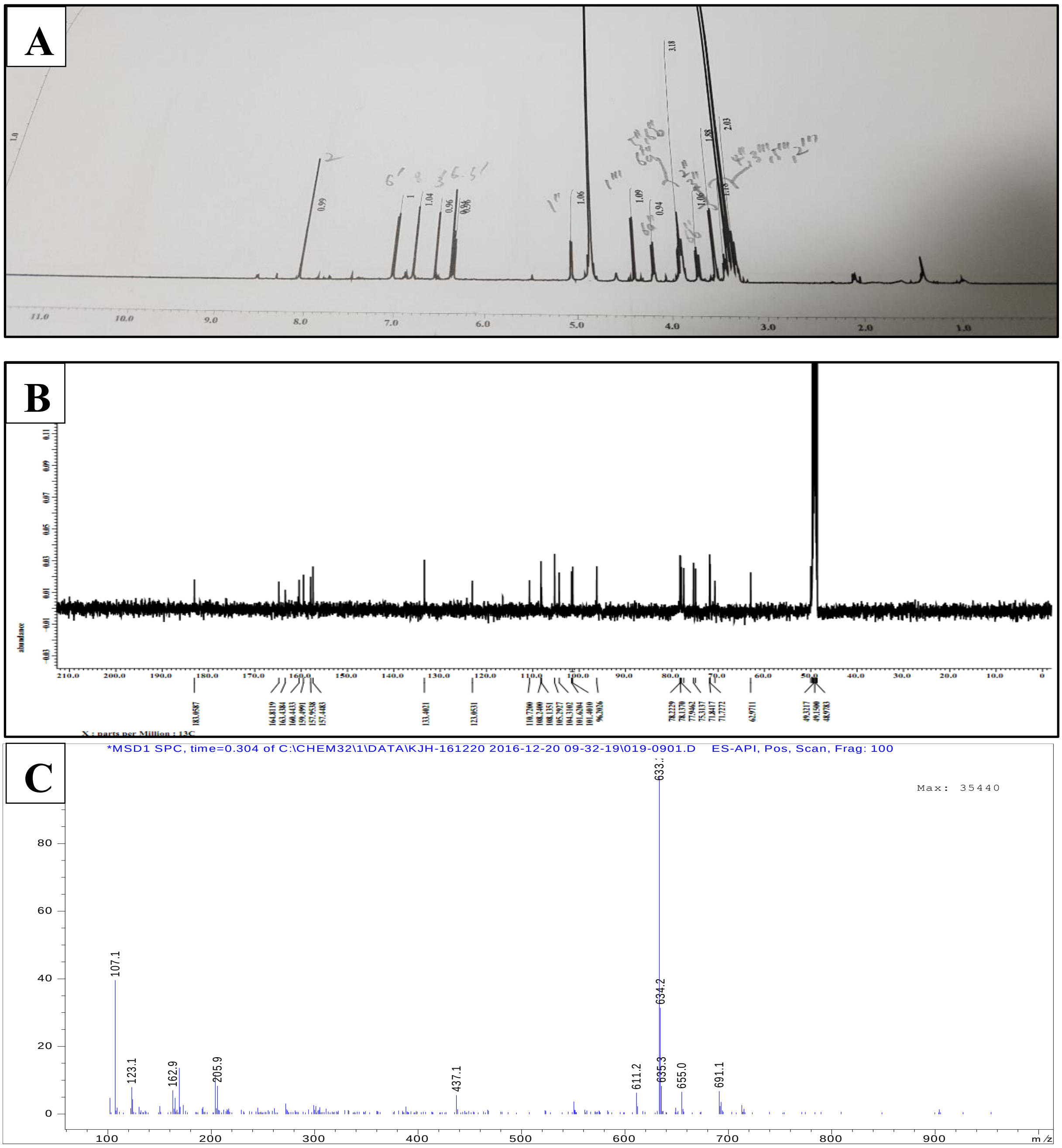

Figure S10.^{1}H(A)-/^{13}C(B)-NMR and MS(C) spectra of compound **8**.

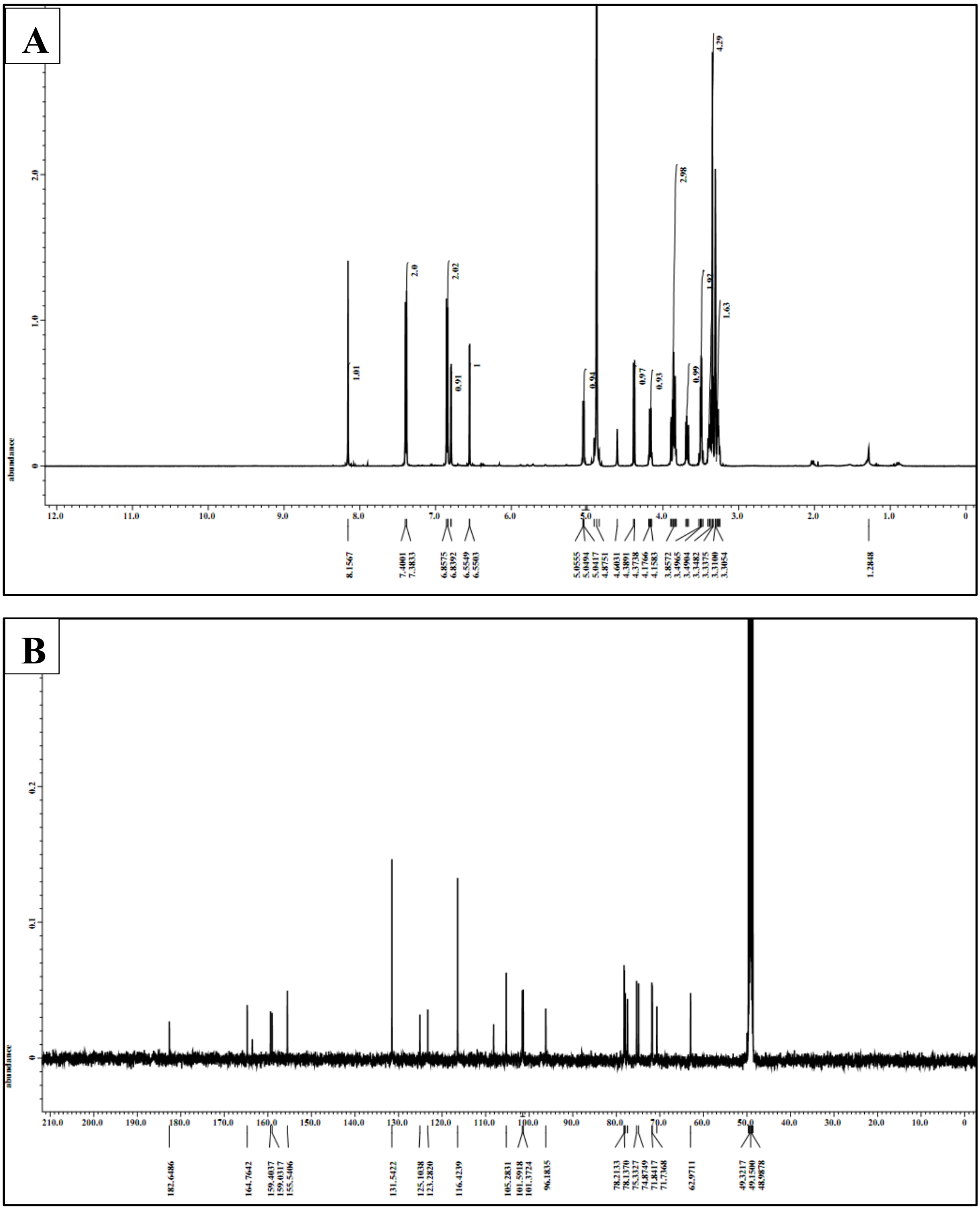

Figure S11. ^{1}H(A) and ^{13}C(B)-NMR of compound **9**.
